# Impact of varied combinatorial mixture of non-fishmeal ingredients on growth, metabolism, immunity and gut microbiota of *Lates calcarifer* (Bloch, 1790) fry

**DOI:** 10.1038/s41598-020-72726-9

**Published:** 2020-10-13

**Authors:** Sanjay K. Gupta, Ravi Fotedar, Md. Javed Foysal, Manisha Priyam, Muhammad A. B. Siddik, Md. Reaz Chaklader, Thi Thanh Thuy Dao, Janet Howieson

**Affiliations:** 1 ICAR-Indian Institute of Agricultural Biotechnology, Ranchi, Jharkhand India; 2 School of Molecular and Life Sciences, Curtin University, Bentley, WA Australia; 3 Department of Genetic Engineering and Biotechnology, Shahjalal University of Science and Technology, Sylhet, Bangladesh; 4 Department of Fisheries Biology and Genetics, Patuakhali Science and Technology University, Patuakhali, 8602 Bangladesh

**Keywords:** Biotechnology, Computational biology and bioinformatics, Immunology, Molecular biology, Physiology, Zoology

## Abstract

The search for suitable fish meal replacements in aqua-diets is a salient agenda in the constant effort of making aquaculture practices more sustainable. In this study, we tested four customised diets composed by systematic inclusion of pre-selected fish meal substitutes, lupin kernel meal, BSF meal, TH and PBM on growth, metabolism, cytokine profile, gut morphology and microbiota of juvenile *Lates calcarifer*. Five isoproteic and isoenergetic diets were prepared viz. FM100 as a control (without fish meal substitute), while FM75, FM50, FM25 and FM0 indicates replacement of fish meal (FM) at 25%, 50%, 75%, and 100%, respectively by a mixture of four different pre-selected non-fish meal (NFM) ingredients. Fish fed FM100, FM75, FM50, FM25 exhibited consistent growth and haematological response, while the fish fed no fishmeal (FM0) showed significant decline in final body weight (FBW) and specific growth rate (SGR). The poor growth performance was correlated with a decrease in villous width, microvilli height and goblet cells density. A significant shift in abundance profile of *Psychrobacter* in the gut microbial profile of fish fed FM50 was noticed compared to fish fed FM100. The results of qRT-PCR showed up-regulated expression of innate immune responsive genes in the FM50 group. The adverse impacts on growth performance and gut health of fish fed FM0 suggest that the complete substitution of fishmeal is not advisable and the inclusion range of these alternatives should be decided for a species only after examining their effect on maximal physiological performance.

## Introduction

Fishmeal (FM) is an exemplary source of dietary protein for aquaculture species due to its high protein content, omega-3 polyunsaturated fatty acid, balanced amino acid composition and easy digestibility^1^. However, the dependence of aquaculture on FM for protein source is a rising concern for this industry with regards to its economics and sustainability. Aquaculturists worldwide have been conducting trials to come up with alternative ingredients for FM that can be cost-effective, easily available and yet provides the same nutritional value for the target species^2,3^. Attempts have been made to exploit both plant and animal protein sources as total or partial FM replacements in aqua-diets to achieve results comparable to diets with FM^4–9^. FM replacement, solely, by plant-based alternatives has been a challenge mainly due to the presence of anti-nutritional factors (ANFs), including alkaloids, lectins, protease inhibitors, phytates, saponins and tannins, deficiency of lysine and methionine and concerns about digestibility and palatability^10^. On the other hand, animal-based sources like poultry by meal (PBM), blood/ bone/ meat meal etc. despite having high protein content and lack of ANFs demonstrated a deficit in lysine, methionine and isoleucine^4^. Researchers are trying to navigate these concerns by exogenous supplementation of essential amino acids or biochemical manipulation of the constituents^10–14^. Inclusion of both plant and animal meal has also exhibited promising results in various species^15,16^. Recycling of marine by-products is another tested alternative for FM. The marine fish processing industry utilises only raw flesh for packaging while about two-thirds of the fish is discarded^17^. As one of the most lucrative species on the international fish market, tuna (*Thunnus spp.*) also contributes to a huge load of by-product. Hydrolysis of skipjack tuna (*Katsuwonus pelamis*) waste provides tuna hydrolysate (TH) which is rich in low molecular weight peptides and free amino acids and holds great potential for economic viability of aquaculture industry^18^. Addition of yellowfish TH to the diet of Persian sturgeon (*Acipenser persicus*) larvae resulted in maximised growth and feed utilisation at 25% FM replacement while showing no impact on gut microflora count or immunity^19^. TH also proved to be effective in improving immunity and dietary protein digestibility in red sea bream (*Pagrus major*) and olive flounder (*Paralichthys olivaceus*)^20^. With high protein and cholesterol content, ubiquitous availability and ease of species palatability, PBM is another promising FM replacement for considerable cost-cutting in aqua-feed production^21,22^. Up to 65% incorporation of PBM has been feasible in channel catfish (*Ictalurus punctatus*) without hampering its growth^23^. A recent study by Sabbagh et al., demonstrated the possibility of total FM replacement by PBM in gilthead seabream (*Sparus aurata*) showing comparable growth performance, fillet quality and haematological parameters to that of fish fed 100% FM^24^. Galkanda-Arachchige et al. performed a meta-study on assessment of PBM as a FM substitute that showed insignificant weight variations between various dietary treatments while significant in-study variations were noted between fish species^25^. Another promising FM substitute is insect meal, especially black soldier fly (BSF) (*Hermetia illucens*) larvae meal that has led to improvement in growth performance and immune indices in yellow catfish (*Pelteobagrus fulvidraco*) at partial incorporation levels^26^. BSF meal has also demonstrated potent results in juvenile Nile tilapia, (*Oreochromis niloticus*) and marron (*Cherax cainii*) but its commercial application still requires consideration on issue of mass production and cost management^27,28^.

*Lates calcarifer* or barramundi, distributed across the Indo-specific and Australia, is a commercially viable species due to its high physiological tolerance, fast growth, easy maintenance and production^29^. As an obligate carnivore, it requires high quality protein for maintenance of its optimum growth. In the recent years, a variety of FM substitutes have been tested on barramundi as well, amongst other marine carnivore species. *L. calcarifer* showed an improvement in feed intake and growth performance with skipjack TH -supplemented diets^30^. Sixty percent inclusion of fermented lupin (*Lupinus angustifolius*) in the diet of juvenile barramundi had no adverse effect on its body weight or fish carcass composition^31^. Replacement of FM was also found to be feasible with *H. illucens* meal in the range of 28.4–50%^32^. Though the results from these studies indicate the potential of these raw ingredients for commercial aqua-diet preparation, yet the majority are focussed on testing the efficacy of a stand-alone plant and animal based protein source. The mixture of various protein sources may be able to meet the nutritional requirements comparable to FM. Hence, there is a need for a thorough assessment of impacts of dietary inclusion of a mixture of raw plant and animal based proteins to replace FM. Reports on implication of gut microbiota profile as a determinant of physiological aspects like digestion and immunity highlight the critical relationship between aquadiets and gut health^33^. With high throughput sequencing technologies becoming advanced and cheaper by the day, the analysis of the data via high processor speed and computational biology algorithms would prove insightful in understanding the regulation of fish physiological systems by outlining gut microbes^34^.

The current study was undertaken to assess the impact of including various concentrations of a combination of non-fish meal ingredients such as lupin kernel, tuna hydrolysate, BSF meal, and PBM on the growth performance, biochemical indices, cytokine profile of distal gut and its microbiota composition. Starting from FM100 which had 100% FM, step by step replacement of FM was done in each of the subsequent diet preparations to formulate FM75, FM50, FM25 and FM0 diets by increasing the variety of non-FM ingredients.

## Results

### Growth parameters

Growth performance, food conversion ratio (FCR), hepatosomatic index (HSI) and survival of barramundi fed with four different non-fish meal experimental diets and the control are shown in Table 1. Significantly lower final body weight (FBW) and specific growth rate (SGR) were observed in the barramundi fed without fish meal than the other dietary treatment groups. Similarly, biomass gain (BG) was significantly decreased in the group fed with completely replaced fish meal fed group (FM0), however, non-significant change was observed compared to control and 25% fish meal substituted group (FM75). FCR and HSI of juvenile barramundi were not affected by any dietary treatments. Although survival of juveniles was the highest in FM50 compared to other treatment groups, but it was non-significant.

$$IBW:Initial\,body\,weight \,({\text{g}});{\text{FBW}}:{\text{Mean}}\,{\text{final}}\,{\text{body}}\,{\text{weight}}\,({\text{g}})$$

$$SGR:Specific\,growth\,rate = (\ln FBW - \ln IBW)/days \times 100$$

$$BG:biomass\,gain = ((final\,biomass - initial\,biomass))/initial\,biomass \times 100$$

$$FCR:Feed\,conversion\,ratio = dry\,feed/ wet\,weight\,gain$$

$$HSI:Hepatosomatic\,index = (weight\,of\,liver/ weight\,of\,fish) \times 100$$

$$Survival\,(\% ) = (no.\, of\,final\,fish - no.\,of\,initial\,fish)/ no.\,of\,initial\,fish \times 100$$

**Table 1:** Growth performance of juvenile barramundi fed with different non-fish meal diets for 7 weeks.

Growth parameters	Experimental diets				
	FM100	FM75	FM50	FM25	FM0
IBW (g)	2.51 ± 0.01	2.53 ± 0.03	2.54 ± 0.02	2.53 ± 0.02	2.60 ± 0.06
FBW (g)	63.70^a^ ± 3.14	63.19^a^ ± 1.96	62.68^a^ ± 2.71	60.72^a^ ± 3.01	44.27^b^ ± 1.55
SGR (%/d)	6.60^a^ ± 0.10	6.56^a^ ± 0.06	6.53^a^ ± 0.09	6.48^a^ ± 0.10	5.79^b^ ± 0.03
BG (%)	1041.91^ab^ ± 68.21	1070.20^ab^ ± 28.06	1231.36^b^ ± 40.35	1136.36^b^ ± 98.87	854.10^a^ ± 19.02
FCR	1.26 ± 0.03	1.28 ± 0.05	1.17 ± 0.02	1.28 ± 0.09	1.53 ± 0.17
HSI	1.69 ± 0.11	1.91 ± 0.16	1.77 ± 0.21	2.19 ± 0.31	2.10 ± 0.17
Survival (%)	81.67^a^ ± 1.67	85.00^ab^ ± 5.00	98.33^b^ ± 1.67	93.33^ab^ ± 4.41	96.67^ab^ ± 3.33

Different superscript letters (a, b) in the same row denote significant differences (p < 0.05). Data were represented as mean ± SE.

### Serum and blood biochemical indices

**Figure 1:**
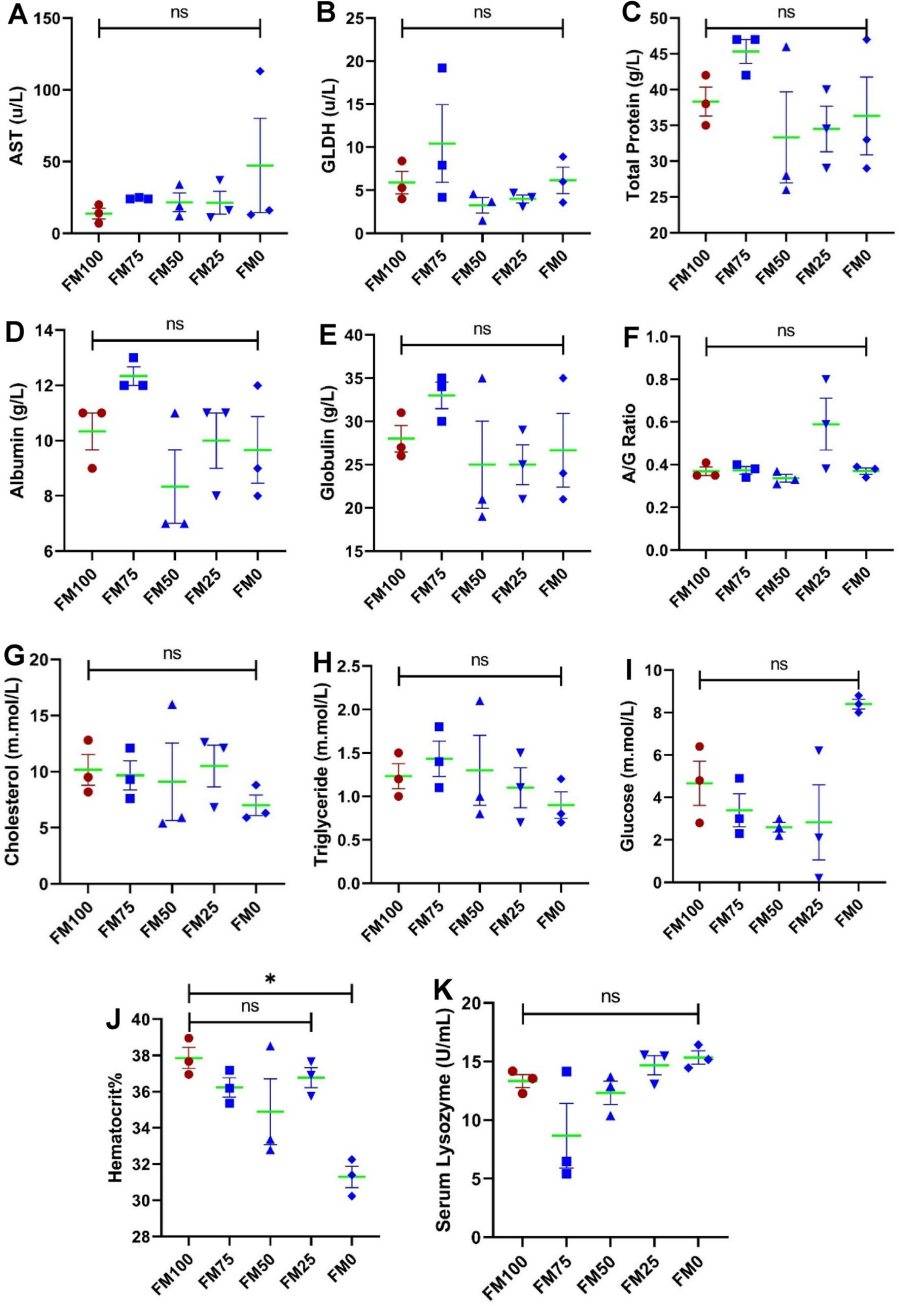
Serum and blood biochemical indices of juvenile barramundi fed with different protein sources for 7 weeks. X-axis represents the different non protein source 0 (control, FM100), 25, 50, 75 and 100 are considered as experimental treatments. (**A**) AST, aspartate transaminase, (**B**) GLDH, glutamate dehydrogenase, (**C**) total protein, (**D**) albumin, (**E**) globulin, (**F**) A/G ratio (albumin/globulin ratio), (**G**) Cholesterol, (**H**) Triglyceride, (**I**) Glucose, (**J**) Heamatocrit % and (**K**) Serum lysozyme. Data were represented as mean ± S.E., n = 3. Post ANOVA Turkey multiple comparison test was applied to compare the mean value of each treatment with the mean value of the control. Mean values significantly different from the control are noted with *P* < 0.05.

Apart from haematocrit % (Fig. 1J) none of the measured blood and serum biochemical indices including, aspartate aminotransferase (AST), glutamate dehydrogenase (GLDH), total protein, albumin, globulin, albumin and globulin ratio (A/G ratio) cholesterol, triglyceride, glucose and serum lysozyme were significantly affected by inclusion of various non-fish meal ingredients in the diets of juvenile barramundi (Fig. 1A–K). Significant decline in the haematocrit level was observed between fish meal (FM100) and non-fish meal (FM0) fed groups, however, non-significant change was noticed among other treatments (Fig. 1J).

### Histomorphology

**Figure 2:**
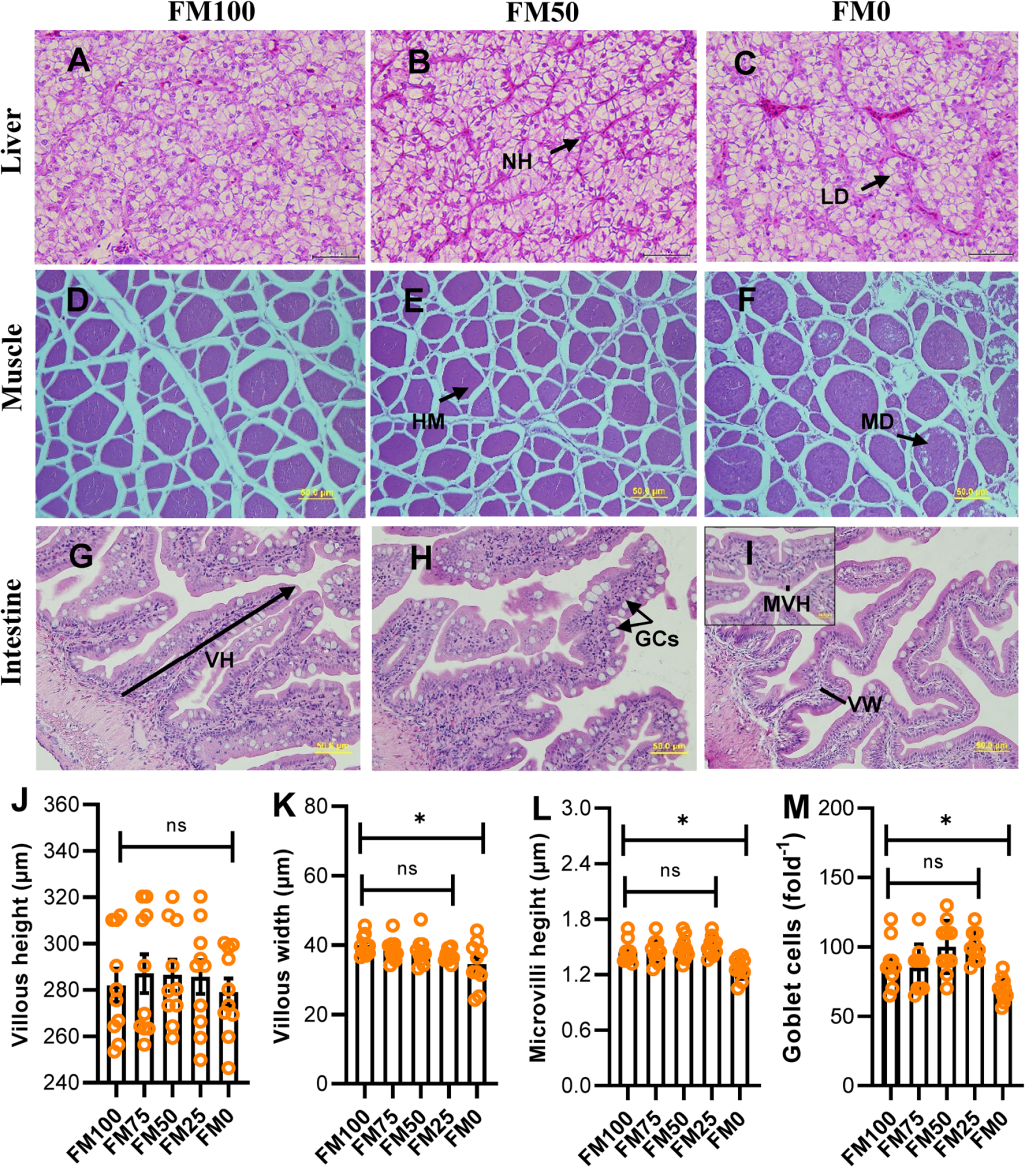
Representative micrographs of liver (**A**–**C**), muscle (**D**–**F**) and intestine (**G**–**I**) of juvenile barramundi after 7 weeks of experimental trials. All sections are stained with H&E with 40 magnification. Variation in villous height (**J**), villous width (**K**), microvilli height (**L**) and goblet cells density (**M**) in fish fed test diets, compared by Turkey multiple comparison test at P < 0.05. NH, normal hepatocyte; LD, lipid droplet; HM, healthy myotome; DM, myodigeneration; VH, villous height; GCs, goblet cells; VW, villous width and MVH, microvilli height.

Histomorphological changes in the liver, muscle and intestinal mucosal morphological examination of juvenile barramundi fed with FM100, FM50 and FM0 diets were demonstrated in Fig. 2. Liver histology of barramundi fed FM100 exhibited increased lipid deposition in hepatocytes while normal cells as indicated by clear hexagonal hepatocytes with prominent nuclei and rare cytoplasmic vacuolization were observed in FM50 fed fish. However, cellular degeneration and necrotic foci were observed in the liver of fish fed FM0. Muscle tissues of juvenile barramundi fed different non FM protein sources showed healthy myotomes characterised by rounded, packed and uniformly identical muscle fibres in fish fed FM50 while necrosis and myodegeneration was observed in fish fed FM0 and FM100. The distal intestine of fish fed FM0 showed notable alteration including reduced villous width, microvilli height and goblet cells density whereas intestinal mucosal morphology were unchanged in fish fed with other treatment diets.

### Alpha diversity of microbial communities

**Figure 3:**
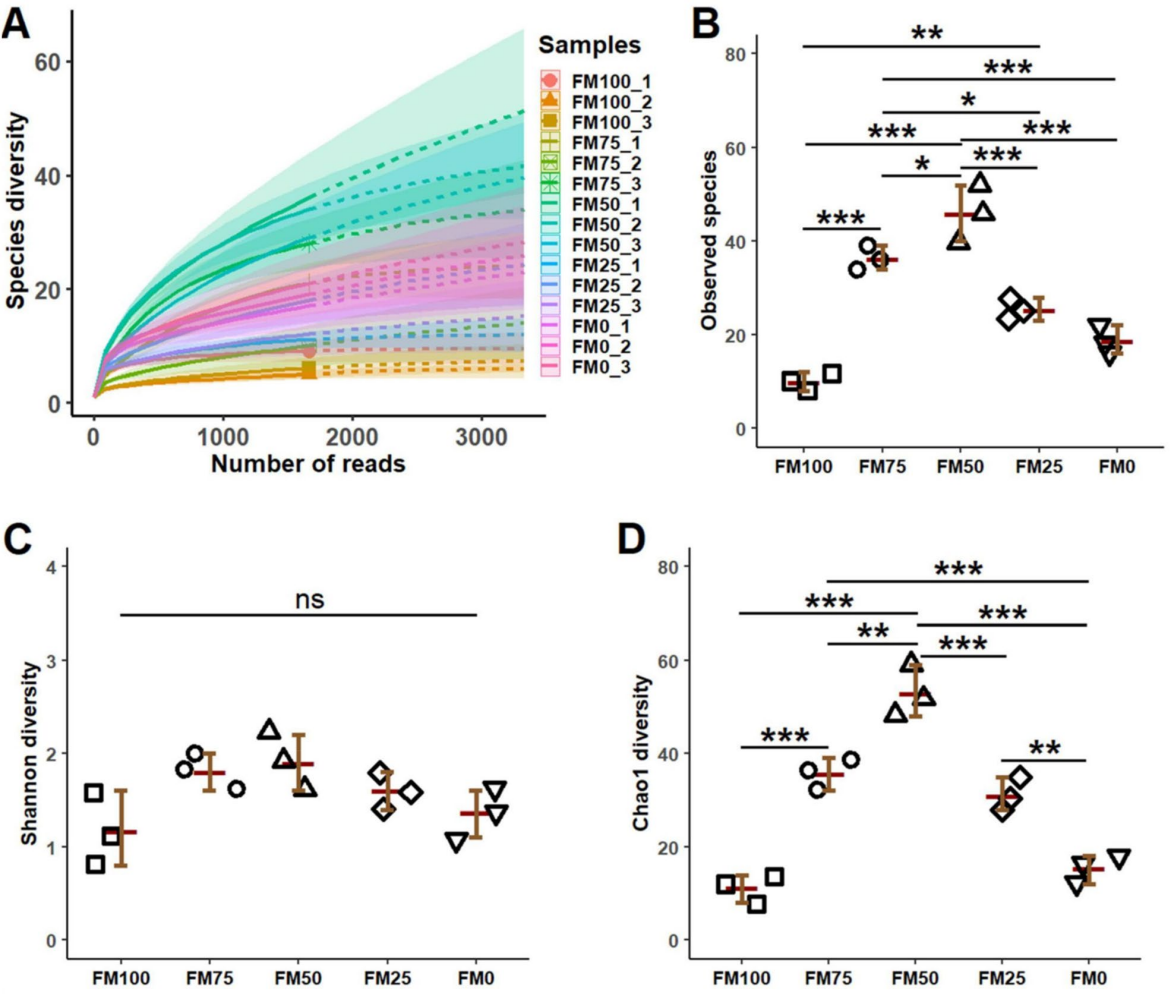
Rare faction curve and diversity indices (mean ± S.E., n = 3) of bacterial genera in juvenile barramundi fed with different non-fish meal diets for 7 weeks of feeding trials. (**A**) Species diversity, (**B**) Observed species, (**C**) Shanon diversity and (**D**) Chaol 1 diversity (ns = Non-significant).

After quality trimming, 15 samples generated 878,788 high quality Illumina pair-end reads that were classified into 125 OTUs, 6 phyla and 56 genera. The rarefaction curve indicated that the 16S rRNA sequence captured enough depth and diversity for 15 samples from five different treatment groups (Fig. 3A). The curve revealed significant influences of bacterial communities in the hindgut of barramundi fed FM50. The alpha diversity measurements revealed that FM50 diet had significant positive influence on observed species and Chao1 indices, followed by FM75, FM25, FM0 and FM100, respectively. The Shannon index on the other hand remained unchanged with all five test diets. The diversity data also showed that FM100 and FM0 diets generated inadequate results in terms of microbial diversity (Fig. 3A–D).

### Beta diversity and microbial communities

**Figure 4:**
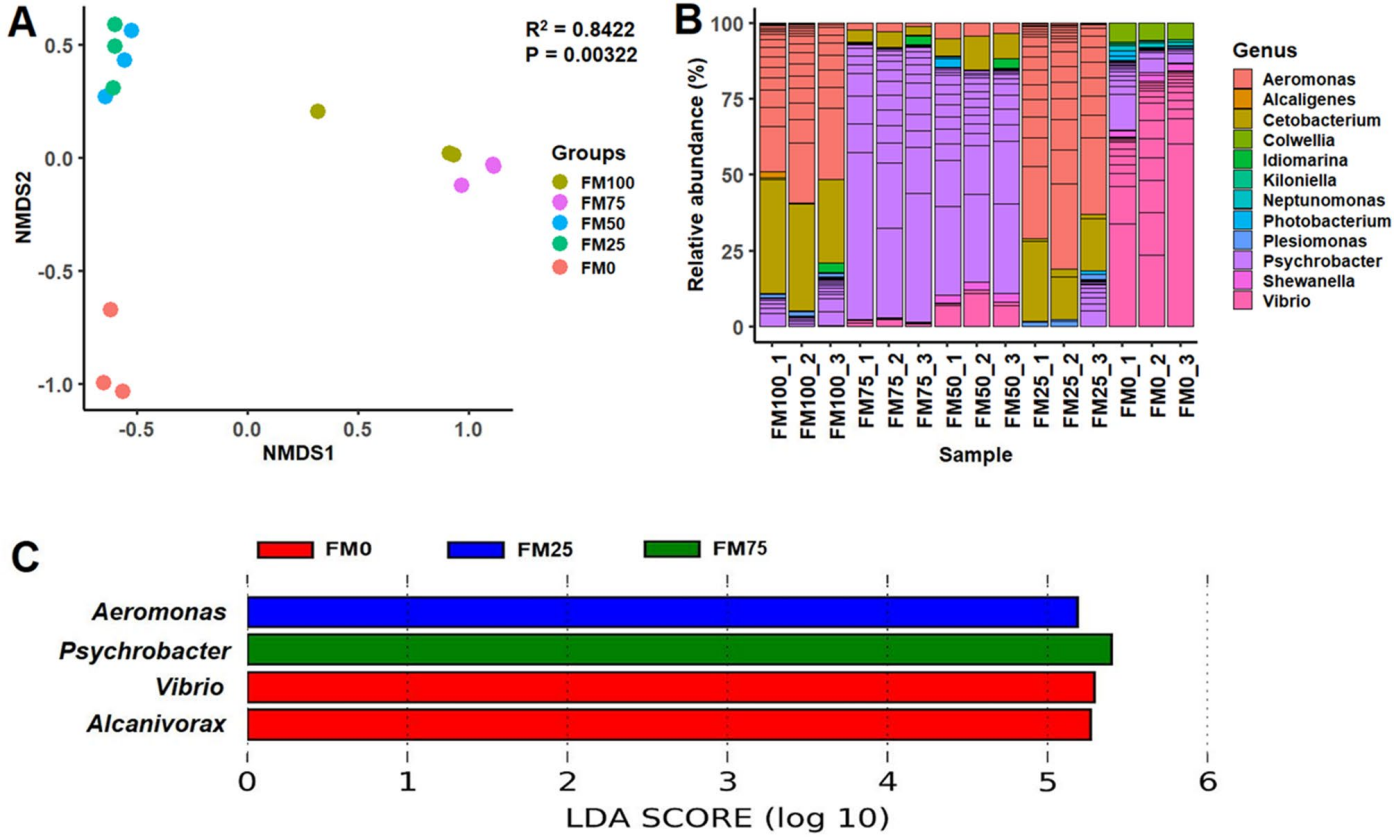
Non-metric multidimensional scaling (nMDS) representing the clustering of samples for five different dietary protein fed groups based on relative abundance of bacterial OTUs (**A**). Relative abundance of bacterial OTUs at Genus level of five different dietary protein fed groups (**B**). Wisconsin non-parametric t-test at 0.05 level of significance and stringent LDA cut-off value of 3.0. (**C**).

Beta dispersion of samples based on non-metric multidimensional scaling (nMDS) is shown in Fig. 4A. An R^2^ value of 0.8422 and P value of 0.00322 showed significant effects of different dietary protein ingredients on barramundi gut. Compare to FM0, FM75 and FM50 had almost same effect while FM25 and FM100 had similar kind of effects on the gut microbiota of juvenile barramundi (Fig. 4A).

At phylum level, Proteobacteria was the most abundant (88.2–98.6%) bacteria in all five diet groups wherein Fusobacteria observed only for FM75 (6.8%) and FM50 (4.8%) (Fig. 4B). At genus level, FM25 (75.9%) and FM100 (48.2%) were dominated by *Aeromonas*, FM75 (88.6%) and FM50 (72.4%) had *Psychrobacter* profusion and FM0 dominated by *Vibrio* (74.6%) (Fig. 3B). The differential abundance at 0.05 level of significance revealed that *Vibrio* and *Alcanivorax* were the indicator microbes in FM0, *Psychrobacter* in FM75 and *Aeromonas* in FM25, respectively (Fig. 4C).

### Impacts on immune gene expression

**Figure 5:**
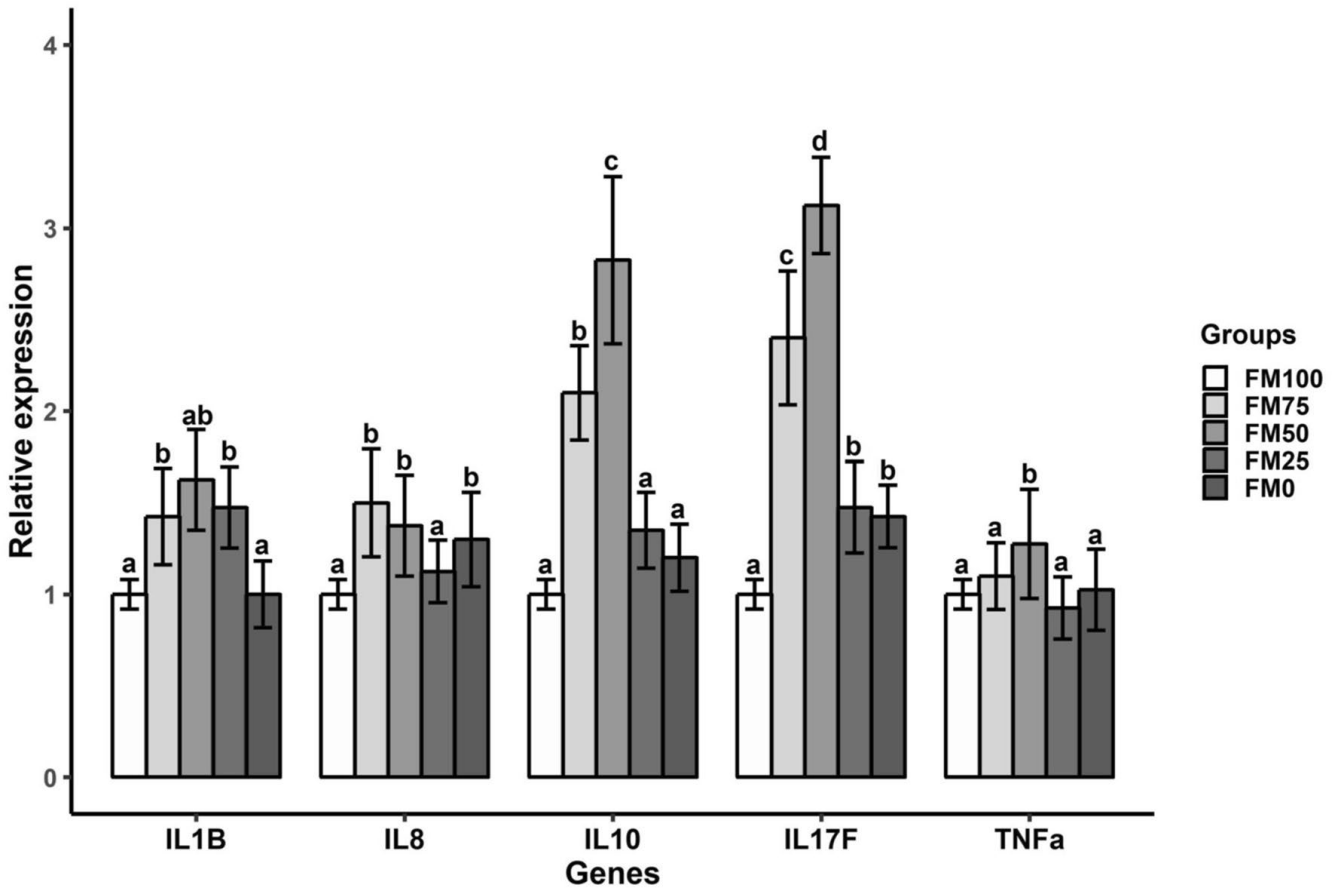
Relative expression level of some cytokine genes in distal intestine of barramundi at the end of 7 weeks feeding trial. Super script letter (a, b and c) on the top of error bar represents significant difference with *P* < 0.05.

Relative to control, the results of qRT-PCR showed up-regulation of innate immune responsive genes in the FM50 group. The expression level of immune genes in the juvenile barramundi fed FM75 had similar patterns to the FM50 group. There was 3.1 and 2.4-fold increase in the expression of pro-inflammatory cytokine, interleukin-17 (IL-17) with FM50 and FM75 diets, respectively (Fig. 5). On the other hand, 2.8 and 2.1-fold increase in the expression of anti-inflammatory cytokine, interleukin 10 (IL10) was observed in the group fed with FM50 and FM75 diets, respectively. Compared to control, 1.6-fold, 1.4-fold and 1.3-fold upregulated expression level of interleukin 1β (IL-1β), interleukin 8 (IL-8) and tumour necrosis factor (TNF-α) genes were observed in FM50 group. The expression of IL-1β, IL10 and TNF-α in the group fed without fish meal inclusion (FM0) was relatively static in compare to control group fed solely with fish meal protein ingredients (FM100).

## Discussion

Multiple studies have been carried out for testing the efficiency of alternatives for FM in *L. calcarifer*^32,35–37^. The choice of raw ingredient is often guided by the ease and cost of availing it in bulk locally, however, with respect to impact assessment, growth parameters should not be the sole criterion for its selection, physiological factors like metabolism, immunity and gut microbial composition should also be taken into account while optimizing the inclusion range of a certain FM substitute. The present study assessed the impact of four experimental diets customised by systematic inclusion of four FM substitutes—lupin kernel meal, BSF meal, TH and PBM on growth, metabolism, immunity and gut microflora of *L. calcarifer* juveniles.

The raw ingredients chosen for this study have been studied earlier individually or in dual combination and have shown significant potential for inclusion in juvenile barramundi diet^36–38^. Upto 45% replacement of fish meal protein could be replaced by lupin meal without compromising growth and protein metabolism parameters^39^. At 10% TH inclusion in the diet of juvenile barramundi, there was a significant increase in the fish’s body weight and SGR^38^. The fish fed on this diet also exhibited improved intestinal health and enhanced resistance to *Streptococcus iniae* infection. BSF-supplemented PBM could replace up to 45% FM in juvenile barramundi while improving growth, gut health and immune parameters^36^. In corroboration with the afore mentioned studies on juvenile barramundi, our study also vividly indicated that the complete replacement of fish meal from the diet of barramundi without adversely impacting its health and physiology is not advisable. The fish fed FM0 containing LKM, PBM, BSF and TH exhibited significant decline in growth parameters and hematocrit %. This dietary group also showed distinct fatty liver with intense vacuoles in the hepatocytes, necrosis and myodegeneration in muscle and a decrease in villous width, microvilli height and goblet cells density. The intestinal micomorphology, in particular, villous width, microvilli height and goblet cells density is related to absorptive surface area of intestine that have been reported to have correlation with digestion and absorption^38, 40^. Such changes in the intestinal micromorphology might have induced the depressed growth of fish fed FM0. In addition,the adverse impact on health parameters in FM0 may be endowed upon the presence of anti-nutritional components in lupin in this diet composition. The lupin meal used in our study was not subjected to any pre-treatment (heat, fermentation), which may have allowed the inherent quantities of phytates and tannins to mediate these ill-effects^31^. For instance, Ilham et al., reported depressed growth performance, lower level of leucocrit, multifocal necrosis in muscle and hepatic steatosis in liver in juvenile barramundi when fed with 25 and 75% of lupin meal over a period of 60 days^41^. Meanwhile, survival rate was significantly higher in FM50 compare to control and there was no adverse effect on weight gain of the other groups except FM0. It is interesting to note that the diet compositions with TH exhibited normal myotome in muscle and improved intestinal histomorphology with no implications on weight gain, except for the last group (FM0) that was composed of LKM along with PBM, BSF and TH. Elevated survival rate in FM50 group and improved histomorphology may be attributed to TH courtesy of its wide array of functional properties particularly presence of greater than 90% of low molecular weight peptides (10 kDa)^42^ that stimulate immune response and disease resistance^17,20^. Despite the health promoting capacity of TH, its inclusion percentage was not enhanced in any of the diets. A previous study by Siddik et al., highlights the negative impacts of excess inclusion of TH in juvenile barramundi diet that include reduced growth performance, feed utilisation and digestibility, cytoplasm vacuolization, necrosis and lipid accumulation in hepatic tissue^43^. Proximate composition analysis showed a considerable increase in ash% with the replacement of FM in each subsequent diet formulation. Previous study on barramundi has highlighted the negative correlation between ash content v/s protein digestibility and mineral utilization^44^.

Amongst the biochemical indices analysed in this study, only haematocrit% seemed to vary significantly between the dietary groups FM100 and FM0. The results conformed with those obtained by Khosravi et al., and Siddik et al., where most of the biochemical parameters were unaffected by inclusion of fish hydrolysates in diets of olive flounder and barramundi, respectively^20,38^. For the quantitative assay of immune genes, FM25 (containing BSF, TH, and PBM) and FM50 (containing TH and PBM) groups showed a similar cytokine profile. The inclusion of BSF in the FM25 diet has led to an enhanced inflammatory response which is in congruence with the findings on BSF + PBM/ BSF + FM supplementation in marron^28^. The inflammatory stimulation (upregulated IL-1β and TNF-α, downregulated IL-10) of fermented PBM + TH (from industrial residues) diet has been recently reported in juvenile barramundi, however IL-8 and IL-17F did not vary significantly in comparison to the control groups^45^. The spike in immunomodulatory effect in FM50 and FM25 groups in this study may be due to the presence of antibacterial peptides in fish protein hydrolysates and BSF^46,47^.

Studies on gut microbiota of *Sparus aurata, Dicentrarchus labrax* and *Onchorhynchus mykiss* have highlighted that gut microflora varies significantly in diversity or composition, depending on the diet^48,49^. Recently, Zheng et al., published a study on the effect of rearing salinity on diversity of gut microbiota of barramundi, reporting *Proteobacteria and Firmicutes* as the most abundant phyla^50^. These two phyla along with Actinobacteria dominate the gut microbial population of fish, with variations in relative compositions^37^. The impact of variation in the diet on the composition of gut microbiota is evident from the results of our study. The phyla composition of our control and experimental groups are in congruence with the results of Siddik et al.’s study on effect of fish protein hydrolysate on juvenile barramundi^37^. Our observations show a 70:30 abundance ratio of *Vibrio: Psychrobacter* in the FM100 fed fish, while the FM50 and FM25 diets are seen to transform this ratio to 10:90, increasing the abundance of *Psychrobacter*. This genus has been associated with secretion of omega fatty acids and metabolites for enhancing immunity and antioxidant status^51^. Therefore, in correlation with Smith et al.’s (2017) study on killfish, this may be an indicator of a healthy gut microflora community in FM50 and FM25 group^51^. In grouper, *Epinephelus coioides*, *Psychrobacter* sp. was shown to improve the immunity as well as gut microbial diversity^52,53^. This may be the possible cause for significant enrichment in microbial diversity index and elevated cytokine levels in FM25 fed group. The gut microbiota profile of FM75 and FM0 showed abundance of *Aeromonas* and *Cetobacterium*. While *Aeromonas* is a commonly found genus in gut of freshwater fish^33^, the abundance of vitamin B12 producing *Cetobacterium* in the two groups is noteworthy which may be attributed to variation in nutritional components of diets^54,55^.

The results of the current study indicate that FM replacement of 50 to 75% could be incorporated in the diet of juvenile barramundi using the appropriate composition of FM substitutes – PBM, BSF and TH. The fish in FM50 and FM25 groups showed a boost in cytokine profile as well as healthy hepatic and intestinal histomorphology. The diets also influenced the gut microbiota composition considerably with enrichment of *Proteobacteria* at the phylum level and *Psychrobacter* at genus level. FM25 also showed significant enrichment of Shannon and Simpson indices in comparison to control. Overall, findings from this study would be cornerstone to ingredient selection for FM replacement in other related carnivore fish^56^, though one has to be mindful of the impact of species-specific diet manipulation on gut microbiota and select the diet composition judiciously^49^. Further extension of this study would be the assessment of bioprocessed non-FM ingredients mixture on physiological parameters of barramundi to see if their inclusion level in the fish diet can be increased.

## Material and methods

### Ethic statements

This experiment was conducted in a recirculating aquaculture system (RAS) facility at Curtin Aquatic Research Laboratory (CARL), Curtin University as per the recommendations of the Guide for the Care and Use of Laboratory Animals of Australia. The study plan and protocols were reviewed and approved by the Ethics Committee in Animal Experimentation of the Curtin University (Approval number ARE2018-33). AQUI-S (8 mg/L) was used as an anaesthetic for proper handling of fish in accordance with the statement of purpose (SOP) of the Curtin Research Laboratories on anaesthetizing fish. Maximum efforts were devoted to alleviate stress and pain for fish for performing all the experimental protocols.

### Test diets

Five isoproteic (crude protein approx. 45%) and isolipidic diets (13% crude lipid) were prepared by encompassing fishmeal, lupin meal, black soldier fly meal, poultry by-product meal and tuna hydrolysate (TH) as the main protein sources. All these ingredients except liquid TH used for preparing experimental diets were supplied by Glen Forest Specialty Feeds, Pvt. Ltd, Perth, Western Australia. Liquid TH was procured from SAMPI, Port Lincoln, Australia. Diet formulations were performed using the Feed LIVE—version 1.52 from Live Informatics Company Limited, Thailand (Table 2).

**Table 2:** Feed ingredients and proximate composition (% dry weight).

Ingredients (% dry weight)^a^	Experimental diets				
	FM100	FM75	FM50	FM25	FM0
Wheat flour	10.13	8.92	6.99	5.92	2.38
Soybean meal	6.99	7.00	7.00	7.00	7.00
Fish meal (FM)	62.98	46.00	30.00	16.00	0.00
Poultry by-product meal (PBM)	0.00	16.00	16.00	16.00	19.00
Tuna hydrolysate (TH)	0.00	0.00	18.00	18.00	18.00
Black soldier fly meal (BSF)	0.00	0.00	0.00	19.00	18.00
Lupin kernel meal (LKM)	0.00	0.00	0.00	0.00	21.24
Wheat Starch	6.99	7.00	7.00	5.00	1.20
Trout Px	0.29	0.29	0.29	0.29	0.29
Canola Oil	3.99	4.00	4.00	4.00	4.00
Fish oil	8.49	8.70	8.70	6.70	6.8
*Sargassum Linearfolium* meal	0.00	2.00	2.00	2.00	2.00
Dicalcium phosphate(DCP)	0.02	0.02	0.02	0.02	0.02
Oxicap E2^b^	0.02	0.02	0.02	0.02	0.02
Stay C (35% Vit C)	0.05	0.05	0.05	0.05	0.05
**Proximate composition (g Kg**^**−1**^** dry weight basis)**					
CP%	44.77	44.63	44.71	44.76	44.59
Lipid%*	12.91	13.08	13.05	13.12	13.05
Fiber%*	0.38	0.77	0.75	0.72	0.65
Ash%*	0.57	1.10	3.12	4.49	5.02

^a^Specialty Feeds, Glen Forrest Stockfeeders, 3150 Great Eastern Highway, Glen Forrest, Western Australia 6071.; CP (Crude protein).

^b^Oxicap E2 contains ethoxyquin, butylated hydroxytoluene, and synergistic chelating agents. *Data were obtained from MixFeed software database program (https://softimbra.com/home).

The raw materials of feed were weighed, crushed and sieved through a 100 μm mesh to obtain fine, uniform sized particles. These ingredients were mixed thoroughly followed by addition of distilled water to the mix to enhance the moisture level for easy pelletizing. The dough was subsequently processed through a pellet extruder, and the pellets were dried in the oven, overnight at 60 °C. The protein content of each of the dietary feeds was confirmed by modifying the formulation until a minima of ± 44.50% was obtained. These diets were labelled as FM100 as a control, FM75, FM50, FM25 and FM0 to replace fishmeal by non-fishmeal protein ingredients at 25% with (PBM), 50% with (25% PBM + 25% TH), 75% with (25% PBM + 25% TH + 25% BSF), and 100% with (25% PBM + 25% TH + 25% BSF + 25% LKM), respectively. Except control (FM100), all the diets were supplied with 2% *Sargassum Linearfolium* meal as mineral supplement source. The pelleted formulations were stored in air tight plastic containers in the feed storage unit until further use.

### Fish husbandry and management

In total, 320 barramundi juveniles were procured from the Mainstream Aquaculture Pty Ltd., Werribee VIC 3030 after size grading. The fish were acclimated for a period of 14 days at Curtin aquaculture research laborarory (CARL) during which they were fed commercially formulated diet (470 g protein kg^−1^ diet and 20.0 MJ kg^−1^ dietary gross energy), twice a day. On the 15th day, 300 juveniles (pool weight of 2.52 ± 0.11 g/ fish) were randomly distributed into 15 independent tanks (300-L water capacity) at a stocking density of 20 fish/ tank. Each tank was equipped with an aerator, electric heater and an external bio-filter (Astro 2212, China). The water quality parameters like temperature (27.60–29.40 °C), salinity (5–6 ppt), dissolved oxygen (5.90–7.51 mgL^−1^), ammonia nitrogen (< 0.50 mg L^−1^) were monitored on daily basis and observed to be within the suitable range for culture of the barramundi fry in recirculating aquaculture systems^57^. The light regime of 14-h light/10-h dark cycle was set using an automatic indoor light switch (Clipsal, Australia).

According to the experimental feed regime, the juveniles in each group were fed respective diets, twice a day at 09:00 and 17:00 h, for a duration of 7 weeks. Feed intake was calculated by subtracting the dry weight of siphoned, uneaten food in the tank from the weight of food provided. The mortality, if any, in each group was recorded during the course of 7 weeks and the weight of dead fish were recorded. The fish were starved for 24 h, and bulk weight and individual weight was recorded for each group of fries for evaluation of growth parameters.

### Biochemical indices of blood and serum

At the end of 7 week of feeding, the fish were anesthetized with AQUI-S (8 mg L^−1^) and blood was drawn from caudal vein of 2 fish per tank (n = 6) using a 1 mL non-heparinized syringe. The blood from one animal was then transferred to a heparinised tube to determine hematocrit level as per the protocol of McLeay and Gordon and expressed as a Hematocrit% (Ht%)^58^. The blood from second animal was transferred to non-heparinised tubes, kept at room temperature until coagulation and finally centrifuged (3000 rpm, 15 min) at 4 °C to obtain serum. The collected serum samples were stored at − 80 °C until further use for determination of hematological parameters. Biochemical indices like triglyceride (TG), cholesterol, glutamate dehydrogenase (GLDH), aspartate aminotransferase (AST), total protein and albumin were determined using an automated blood analyzer (SLIM; SEAC Inc, Florence, Italy) in accordance with the protocols from Blanc et al.^59^. The albumin and globulin ratio (A/G ratio) was calculated by dividing the total albumin content by the difference of total serum and albumin protein values. Cholesterol and TG content was determined according to the method of Siddik, et al.^38^. For recording serum lysozyme, the previously described protocol of Le and Fotedar^60^ was followed.

### Histomorphology

Liver, muscle and distal intestine were excised from ten sacrificed fish in each treatment group (two fish/replicate) and processed for histological analysis. The excised tissues were fixed in 10% neutral buffered formalin before dehydrating them in graded alcohol dilutions. Tissues were treated with xylene before embedding in paraffin wax. Wax-embedded tissue blocks were sectioned at 5 µm on a rotary microtome. The sections were processed for hematoxylin and eosin (H&E) staining following the standard histological protocols. The sections were scanned under a light imaging microscope (BX40F4, Olympus, Tokyo, Japan) for obtaining digital photographs. Intestinal mucosal morphology in terms of villous height, villous width, microvilli height and goblet cells density were measured and quantified randomly from ten intact villi following our earlier studies^38,61^.

### RNA extraction and qRT-PCR analysis

After collection of blood from the anesthetized fish, they were euthanized (AQUI-S, 175 mg l^−1^) (two/replicate) and distal gut was excised and stored in RNA Later (Sigma-Aldrich, Germany) at − 80 °C until RNA extraction. The tissue (~ 5 mg) was thawed and homogenised to a fine powder before processing for RNA extraction as per the manufacturer’s instructions RNeasy Mini Kit (Qiagen, Hilden, Germany). DNase treatment was carried out using RNase free DNase-I (Qiagen, Hilden, Germany) before RNA QC via agarose gel electrophoresis and NanoDrop spectrophotometer 2000c (Thermo Fisher Scientific, USA). cDNA was prepared from 1 µg of RNA as per the manufacturer’s protocol of Omnicript RT kit (Qiagen, Hilden, Germany).

qPCR for IL-1β, IL-8, IL-10, IL-17F, and β-actin was carried out using cDNA samples of the distal intestine in 7500 Real-Time PCR System (Applied Biosystems, USA) employing PowerUp SYBR Green Master Mix (Thermo Scientific, USA) as per the manufacturer’s protocols after standardization. The total volume of PCR reaction was 20 µl consisting of 10 μl TransStart Top Green qPCR SuperMix (2 ×), 0.6 μl of each primer, 1 μl cDNA, and 7.8 μl RNase-free H_2_O, 4 μM of each forward and reverse gene-specific primer (Table 3). geNorm (v3.5) was used to normalize the quantitative real-time PCR data and the expression levels of IL-1β, IL-8, IL-10, IL-17F were calculated using 2 ^−△△CT^ method. One-way ANOVA with Tukey’s HSD was used to compare the mean ± SE of relative expression among the treatment groups.

**Table 3:** List of gene specific primers used for qRT-PCR analysis.

Primers	Sequence (Forward, 5ʹ–3ʹ)	Sequence (Reverse, 5ʹ–3ʹ)	References
IL-1β	ACAACGTCATGGAGCTCTGG	TCTTTGTCCTTCACCGCCTC	Zoccola et al.^71^
IL-8	GTCTGAGAAGCCTGGGAGTG	GCAATGGGAGTTAGCAGGAA	Miao et al.^72^
IL-10	CGACCAGCTCAAGAGTGATG	AGAGGCTGCATGGTTTCTGT	Miao et al.^72^
IL-17F	GTCTCTGTCACCGTGGAC	TGGGCCTCACACAGGTACA	Miao et al.^72^
TNF-α	GCCATCTATCTGGGTGCAGT	AAAGTGCAAACACCCCAAAG	Zoccola et al.^71^
β-actin	CTTCACCACCACAGCCGAGA	TGCCGATGGTGATGACCTGT	Alhazzaa et al.^73^

### DNA extraction, 16S amplification and high-throughput sequencing

For DNA extraction, two randomly picked fish from each tank (n = 6) were selected, followed by careful excision of gut inside biological safety hood, and separation of distal gut. The samples were then homogenized in Qiagen Tissue LyserII (Qiagen, Hilden, Germany) and two distal gut samples from each respective tank were pooled together, and transferred to 1.5-mL Eppendorf tube for DNA extraction^62^. Total bacterial DNA was extracted from the pooled distal intestine sample using commercial DNeasy Blood and Tissue Kit (Qiagen, Hilden, Germany) following the manufacturer’s instructions. Nano-Drop spectrophotometer 2000c (Thermo Fisher Scientific, Waltham, CA, USA) was used to measure the concentration of DNA. To make the final concentration to 30 ng/µL, the extracted DNA was subsequently diluted with nuclease-free water. V3-V4 hypervariable region of bacterial 16S was amplified using PCR in a 50 µL reaction mix consisting of 2µL of template DNA, 1µL each of forward and reverse primers, 25 µL of Hot Start 2X Master Mix (New England BioLabs Inc., Ipswich, MA, USA) and 21 µL of nuclease-free water. BioRad S1000 Gradient Thermal Cycler (Bio-Rad Laboratories, Inc., Foster City, California, USA) was used for 30 cycles of amplification reactions. The products were resolved on 1% agarose gel and purified using AMPure beads methods, followed by library preparation and secondary PCR amplicon barcoding according to the Illumina standard protocol (Part # 15044223 Rev. B)^63^. Sequencing (~ 50,000 reads) of each sample was carried out on Illumina MiSeq platform (Illumina Inc., San Diego, California, USA), using Illumina MiSeq v3 kits (600 cycles, Part # MS-102-3003).

### Bioinformatics

The quality of Illumina Miseq generated data was checked using FastQC pipelines^64^. The reads were trimmed to meet quality parameters (–q20 –l 200) using Sickle^65^. Overlapping pair-end sequences were merged using MeFiT pipeline with default parameters^66^. MICCA(version1.7.0)^67^ was used for filtering the chimeric sequences and de novo assembly of 16S rRNA sequences into Operational Taxonomic Units (OTUs) at 97% similarity threshold. The taxonomic classification of detected OTUs was carried out at 97% similarity check against the SILVA database (SILVA 132 release) (www.arb-silva.de)^68^. QIIME (version 1.9.1) and R packages (Verson V3.5.1) (https://www.r-project.org/) were used for calculating the microbial diversity in each sample at a rarefaction depth value of 3696 bp^69^. The calculation of alpha diversity was performed in terms of observed species, Shannon and chao1 indices. ANOSIM was used for the non-parametric statistical analysis of the distance metric at 1000 permutations. Clustering of sample in terms of non-metric multi-dimensional scaling (NMDS) was performed using Bray–Curtis  dissimilarity of weighted UniFrac distance metric. Linear discriminant analysis (LDA) with default parameters was used to identify the differential abundance of bacterial genera in different treatment groups^70^.

### Statistical analysis

Statistical analysis of the data was performed using SPSSfor Windows version 25, IBMCurtin University, Australia. The results of growth performance, serum and blood biometry and immune indices were expressed as mean ± standard error, and checked for normality and homogeneity of variances with Shapiro–Wilk’s and Levene’s tests. Post confirming these two tests, an ordinary one-way analysis of variance (ANOVA) with Tukey’s multiple comparisons test was used to test the significant difference at 0.05 < P < 0.001 where diet was used as an illustrative variable.

## Data Availability

Altogether datasets produced through the study have been presented in the form of figures and tables but are accessible from the corresponding author on considerable demand. The gut microbiome data is available on NCBI database under BioProject No.- PRJNA608700.
